# A novel mutation (p.Glu1389AspfsX16) of the phosphoinositide kinase, FYVE finger containing gene found in a Japanese patient with fleck corneal dystrophy

**Published:** 2012-12-12

**Authors:** Satoshi Kawasaki, Kenta Yamasaki, Hiroko Nakagawa, Katsuhiko Shinomiya, Mina Nakatsukasa, Yoshihide Nakai, Shigeru Kinoshita

**Affiliations:** 1 Department of Ophthalmology, Kyoto Prefectural University of Medicine, Kyoto, Japan; 2 Department of Biomedical Engineering, Faculty of Life and Medical Sciences, Doshisha University, Kyoto, Japan; 3 Tokai Eye Clinic, Tsu, Japan

## Abstract

**Purpose:**

The phosphoinositide kinase, FYVE finger containing (*PIKFYVE*) gene has been identified as a gene responsible for fleck corneal dystrophy (FCD). The purpose of this study is to report a novel mutation of the *PIKFYVE* gene in a Japanese patient with fleck corneal dystrophy.

**Methods:**

Slit-lamp microscopy, corneal topography, and optical coherence tomography were performed for the clinical examination of the patient’s eye. For genetic analysis, peripheral blood was obtained from the patient and her sister. DNA was extracted from the blood and subjected to mutation analysis by sequencing of the *PIKFYVE* gene. The sequencing results were validated with a PCR-fragment length polymorphism analysis.

**Results:**

A 63-year-old woman presented at our clinic with complaints of decreased vision and metamorphopsia in her right eye occurring 1 month before presentation. Both eyes exhibited small, dot-like, white flecks scattered throughout all layers of the corneal stroma, which corresponds to the typical FCD phenotype. The opacities were relatively dominant at the peripheral region of the cornea, yet were found throughout the entire cornea. Sequence analysis revealed that the patient has a heterozygous c.4166_4169delAAGT mutation located at exon 24 of the *PIKFYVE* gene that may cause p.Glu1389AspfsX16 flame-shift mutation, which has never before been reported for FCD.

**Conclusions:**

To the best of our knowledge, this is the first study to show that a novel mutation (p.Glu1389AspfsX16) causing the truncation of the *PIKFYVE* protein causes fleck corneal dystrophy in the Japanese population.

## Introduction

The cornea is one of the most transparent and non-vascularized tissues in the human body, and several active genes [1,2] are thought to be involved in maintaining the homeostasis of the cornea. Recent advances in molecular biology techniques have allowed the genes responsible in most hereditary corneal dystrophies to be identified, including transforming growth factor, beta-induced (TGFBI)-related corneal dystrophies (i.e., granular corneal dystrophy, lattice corneal dystrophy type I, granular corneal dystrophy type 2 (Avellino corneal dystrophy), Reis-Bücklers corneal dystrophy, and Thiel-Behnke corneal dystrophy) [3], Meesmann corneal dystrophy [4,5], macular corneal dystrophy [6], gelatinous drop-like corneal dystrophy [7], and Fuchs’ endothelial dystrophy [8].

Fleck corneal dystrophy (FCD, Online Mendelian Inheritance in Man (OMIM) #121850) was first reported in 1957 by Francois and Neetens [9], and is one of the hereditary corneal dystrophies in which the causative genes have already been identified. This corneal dystrophy is a rare autosomal dominant disease characterized by numerous tiny, dot-like white flecks scattered in all layers of the corneal stroma. Typically, the stroma located in between the flecks is clear, and the endothelium, the epithelium, Bowman’s layer, and Descemet’s membrane are normal. Patients are usually asymptomatic with normal vision, yet a small number of patients report the sensation of a minor photophobia. The flecks in FCD can appear as early as at 2 years of age, or sometimes even at birth, and appear not to progress significantly throughout life [10,11]. Histologically, the corneal flecks appear to correspond to abnormal keratocytes swollen with membrane-limited intracytoplasmic vesicles containing complex lipids and glycosaminoglycans [12]. It has been reported that there are no extracellular abnormalities [12].

In this study, we report a case of FCD bearing a heterozygous flame-shift mutation within the phosphoinositide kinase, FYVE finger containing (*PIKFYVE*) gene. The patient had no obvious vision loss or any complaints related to this corneal dystrophy, and the appropriateness of our identified mutation as a causative one for FCD is theoretically discussed.

## Methods

### Measurement of corneal irregularity and higher-order aberration

Corneal irregularity and higher-order aberration in the patient were investigated using a commercially available corneal topography device (KR-1W; Topcon Corp., Tokyo, Japan).

### Optical coherence tomography of cornea

An optical section of the patient’s cornea was obtained using a commercially available optical coherence tomography (OCT) device (Cirrus HD-OCT; Carl Zeiss Meditec Co. Ltd., Tokyo, Japan).

### Mutation analysis

A 63-year-old woman presented at our clinic with complaints of decreased vision and metamorphopsia in her right eye occurring 1 month before presentation. Her best-corrected visual acuity was 0.7 in her right eye and 1.2 in her left eye. The decreased visual acuity and metamorphopsia seemed to be due to a transient focal retinal detachment caused by the traction of the posterior vitreous membrane.

All experimental procedures were approved by the Institutional Review Board for Human Studies of Kyoto Prefectural University of Medicine. This study was performed in accordance with the tenets of the Declaration of Helsinki for research involving human subjects.

Peripheral blood was obtained from the patient and her younger sister, the patient’s only remaining living relative using a plastic syringe attached with a 23G needle. Prior informed consent was obtained from both subjects after a detailed explanation of the study protocols. Genomic DNA was extracted from the blood using a commercially available kit (DNeasy Blood & Tissue Kit; Qiagen GmbH, Hilden, Germany). Genomic DNA samples from 96 normal Japanese volunteers (48 men and 48 women) were obtained from a research-resource bank (Human Science Research Resource Bank, Osaka, Japan). Using 10 ng of genomic DNA, all exons of the *PIKFYVE* gene were amplified with polymerase chain reaction (PCR) in a 50 μl reaction buffer containing 1 x ExTaq buffer, 0.2 mM dNTP, 0.2 μM primer pair, and 1.25 U Taq polymerase (ExTaq Hot Start version; Takara Bio Inc., Otsu, Japan). All primer pairs were designed according to a previous study [13]. The PCR products were treated with a mixture of exonuclease and alkaline phosphatase (ExoSAP-IT; GE Healthcare UK, Ltd., Buckinghamshire, UK), heat-inactivated, and sequenced using a commercially available kit (BigDye 3.1; Applied Biosystems Inc., Foster City, CA). The sequencing products were purified with a commercially available kit (BigDye Xterminater Purification Kit; Applied Biosystems), electrophoresed on an automated sequencer (3130×l Genetic Analyzer; Applied Biosystems), and analyzed with sequence alignment software (Variant Reporter Version 1.0; Applied Biosystems). Thermal cycle conditions for all primer pairs were 30 cycles of three-temperature thermal cycles at 94 °C for 30 s for heat denaturation, at 55 °C for 30 s for annealing, and 72 °C for 30 s for extension.

### Polymerase chain reaction–fragment length polymorphism analysis

Sequencing data were validated with PCR–fragment length polymorphism (PCR–FLP). Briefly, a partial sequence of exon 24 of the *PIKFYVE* gene was amplified by PCR using a primer pair (PIKFYVE_FLP_F_Ex24; 5′-CTC AGT TAT TCT CCC ATT CGG CTT C-3′, PIKFYVE_FLP_R_Ex24; 5′-AAT GAA TAT TTT GGG GAG TGG AAC A-3′). The PCR product was electrophoresed on a 10% acrylamide gel. After the electrophoresis, the gel was stained with a DNA-staining fluorescent dye (SYBR^®^ Green I; Takara Bio), observed on a UV transilluminator, and photographed in a dark box equipped with a charge-coupled device (CCD) camera (LAS-3000 UV mini; GE Healthcare UK).

## Results

**Figure 1:**
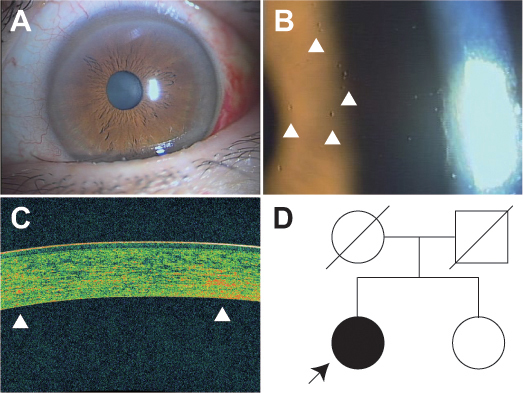
Images demonstrating the corneal phenotypes of a patient with fleck corneal dystrophy. **A**: Stromal flecks are not perceptible under diffuse illumination in both of the patient’s eyes. **B**: Under iris retroillumination, stromal flecks (arrowheads) became evident. **C**: Optical coherence tomography analysis successfully detected the stromal flecks as slightly bright small areas (arrowhead). **D**: The pedigree of the patient with fleck corneal dystrophy is demonstrated.

Both eyes exhibited small, dot-like, white-fleck opacities scattered in all layers of the corneal stroma. The opacities were relatively dominant at the peripheral region of the cornea, yet were found throughout the entire cornea. The opacities were almost invisible under diffuse illumination (Figure 1A), but became more apparent under slit-lamp illumination or iris retroillumination (Figure 1B). It seems difficult to recognize the opacities in ordinary care, especially for ophthalmologists unfamiliar with such faint corneal opacity. OCT analysis successfully identified some of the small stromal flecks (Figure 1C). Higher-order aberration was within the normal limit in both corneas. Specular microscopy examination demonstrated that the endothelial cell density was 2,000 cells/mm^2^ in her right cornea and 2,200 cells/mm^2^ in her left cornea, which is sufficient for endothelial function but appears slightly decreased compared to the average cell density in persons of her same age. She had previously undergone clinical examination by an ophthalmologist several times in her life; however, it was never pointed out to her that she had such corneal abnormalities. Her sister did not exhibit any corneal manifestations in either eye (Figure 1D).

No treatment was undergone by this patient, but her retinal problem spontaneously ameliorated as judged by OCT findings along with the improvement of visual acuity from 0.7 to 1.2 in 2 weeks, indicating that the decrease in the visual acuity of her right eye at her first visit was not due to the corneal opacities.

### Mutation analysis

**Figure 2:**
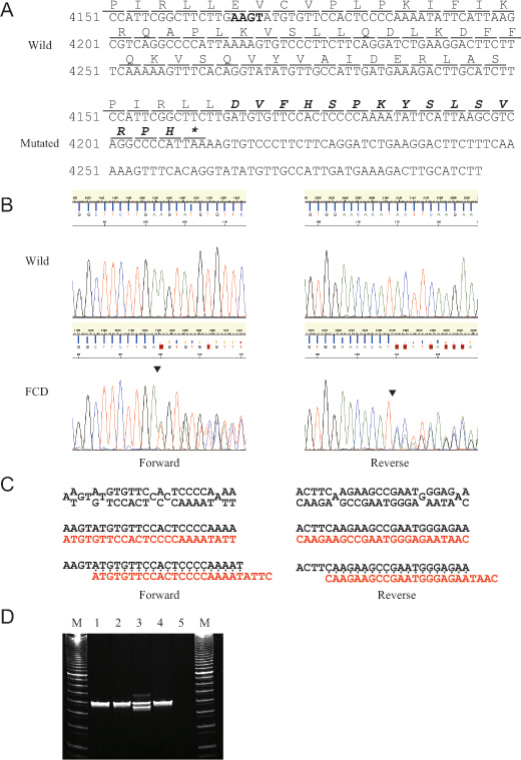
Results of sequencing and polymerase chain reaction (PCR)-fragment length polymorphism (PCR-FLP) analyses for the patient with FCD are demonstrated. **A**: Nucleotide and amino acid sequence of the wild (upper) and mutated (lower) *PIKFYVE* gene around the identified p.Glu1389AspfsX16 mutation are demonstrated. The deleted four bases of the c.4166_4169delAAGT mutation are indicated in bold type in the wild-type sequence. The altered amino acid sequence downstream of the deleted four bases is indicated in bold italics in the mutated sequence. Asterisk (*) means an ochre (TAA) stop codon. **B**: Results of sequencing analysis for exon 24 of the *PIKFYVE* gene in normal volunteer (upper) and the patient with FCD (lower) from forward (left) and reverse (right) directions are demonstrated. Arrowheads indicate the breakpoint of the c.4166_4169delAAGT mutation. **C**: The mixed base sequence (upper) downstream of the breakpoints was subtracted (middle) from the reference sequence (black type) to extract the mutated sequence (red type) in both directions (left: forward, right: reverse). Note that the mutated sequence is fully matched to the reference sequence from four bases downstream of the breakpoints (lower), indicating that the mutated sequence is deleted with four bases, AAGT sequence. **D**: Results of PCR-FLP analysis for exon 24 of the *PIKFYVE* gene in normal volunteers (lanes 1 and 2) and the patient with FCD (lane 3) and her sister (lane 4) are demonstrated. Lane 5 means negative control. Note that the shorter PCR band in the patient with FCD (lane 3) was amplified from the mutated allele while the longer PCR band was from the wild-type allele.

The sequence data revealed that the patient had a heterozygous 4-base-pair deletion mutation (c.4166_4169delAAGT or c.4167_4170delAGTA) within the *PIKFYVE* gene (Figure 2A-C). This mutation may produce a new reading flame starting from amino acid number 1389, leading to a premature termination at the 16^th^ codon counted from the first affected amino acid (p.Glu1389AspfsX16), which has never been reported in patients with FCD. PCR-FLP analysis confirmed these sequencing data (Figure 2D). In addition to this mutation, 20 nucleotide changes were found within the *PIKFYVE* gene (Table 1), yet all were of known single-nucleotide polymorphisms, of synonymous amino acid alteration, or located at the non-coding region, and hence are considered non-pathological. The c.4166_4169delAAGT mutation was not found in any of the examined 96 normal Japanese volunteers (data not shown). The sister of the patient did not have the c.4166_4169delAAGT mutation.

**Table 1:** List of nucleotide changes identified in our FCD patient.

#	Region	Nucleotide change	Zygosity	Type of mutation	Effects on amino acid	SNP
1	Intron 9	g.32610C>T	Homozygous	Substitution	none (non-coding)	none
2	Intron 15	g.48601A>G	Homozygous	Substitution	none (non-coding)	none
3	Exon 16	c.2087G>A	Homozygous	Substitution	p.696S>N	rs10932258
4	Exon 16	c.2106C>T	Homozygous	Substitution	p.702p>P	rs10932259
5	Exon 19	c.2795T>C	Homozygous	Substitution	p.932L>S	rs2363468
6	Exon 19	c.2984A>T	Homozygous	Substitution	p.995Q>L	rs893254
7	Exon 19	c.2993C>G	Homozygous	Substitution	p.998T>S	rs893253
8	Exon 19	c.2984A>T	Homozygous	Substitution	p.995Q>L	rs893254
9	Exon 19	c.2993C>G	Homozygous	Substitution	p.998T>S	rs893253
10	Exon 19	c.3547C>A	Homozygous	Substitution	p.1183Q>K	rs1529979
11	Exon 19	c.3564T>C	Homozygous	Substitution	p.1188n>N	rs1529978
12	Exon 24	c.4166_4169delAAGT	Heterozygous	Insertion	p.Glu1389AspfsX16	none
13	Intron 27	g.65496T>C	Homozygous	Substitution	none (non-coding)	none
14	Intron 31	g.73584G>A	Homozygous	Substitution	none (non-coding)	none
15	Intron 32	g.73754C>T	Homozygous	Substitution	none (non-coding)	none
16	Exon 34	c.5334G>A	Homozygous	Substitution	p.1778T>T	rs2304545
17	Exon 35	c.5397A>G	Homozygous	Substitution	p.1799T>T	rs2118297
18	Intron 35	g.79205A>G	Homozygous	Substitution	none (non-coding)	none
19	Exon 36	c.5526A>G	Homozygous	Substitution	p.1842E>E	rs994697
20	Exon 38	c.5727G>T	Heterozygous	Substitution	p.1909A>A	none
21	Intron 39	g.82947A>G	Homozygous	Substitution	none (non-coding)	none

The notation convention of the nucleotide and protein changes follows the nomenclature guidelines of human genome variation society (HGVS).

## Discussion

In 2003, Jiao et al. [14] performed linkage analysis of four families with FCD and found that the critical region for FCD mapped to a 27.9 cM region of chromosome 2q35 flanked by the genomic markers D2S117 and D2S126. Subsequently, in 2005, Li et al. [15] further narrowed the linked region to a 24 cM interval containing 18M bases. Li et al. subsequently sequenced genes included within the narrowed region and found mutations in the *PIKFYVE* gene in patients with FCD. The *PIKFYVE* gene encodes a widely expressed, 2,089-amino-acid-long, phosphoinositide 3-kinase family member that functions in post-Golgi vesicular sorting [15].

**Figure 3:**
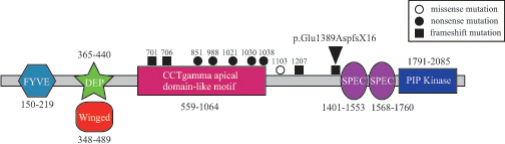
Schematic representation of the domain structure of the *PIKFYVE* gene. Mutations reported thus far are depicted with amino acid numbers and effects on the protein sequence (empty circle: missense, solid circle: nonsense, solid square: frameshift).

In the present study, we found a heterozygous c.4166_4169delAAGT mutation within the *PIKFYVE* gene in our patient with FCD. As this mutation is of 4-base-pair deletion, the mutation may cause a flame-shift amino acid alteration and have a significant impact on the function of the *PIKFYVE* protein. A homology search of its amino acid sequence identified six putative functional domains in the *PIKFYVE* protein [15,16], including a zinc-finger-containing phosphoinositide kinase (FYVE) located at the 150–219 amino acid region, Disheveled, EGL-10, plextrin homology domain (DEP) of unknown function located at the 365–440 amino acid region, a cytosolic chaperonin CCT gamma apical domain-like motif located at the 667–843 amino acid region, a common kinase core motif found in the type IIβ phosphatidylinositol-4-phosphate 5-kinase (PIP kinase) located at the 1791–2085 amino acid region, a small β-sheet “winged helix ” DNA/RNA-binding motif (Winged) located at the 348–489 amino acid region, and two spectrin repeats (SPEC) located at the 1490–1538 and 1679–1729 amino acid regions (Figure 3). Our mutation may lead to a truncation of the *PIKFYVE* protein lacking the two spectrin repeats and phosphatidylinositol-4-phosphate 5-kinase core domains. Thus, the mutation might imply the necessity of at least each of the two domains for completing the function of the *PIKFYVE* protein, at least in corneal keratocytes. The fact that the mutation was not found in any of the examined 96 normal Japanese volunteers, along with the fact that the phenotype well cosegregated with genotype in our pedigree, supports the pathogenicity of the mutation.

There are several genetically modified model organisms for the *PIKFYVE* gene. In *C. elegans* [17] and *Drosophila* [18], several mutant lines were generated for the *PIKFYVE* gene with the ethyl methanesulfonate mutagenesis technique. In both organisms, mutant lines harboring loss-of-function mutations of the gene displayed numerous vacuoles in their cells and died at the embryonic or pupal stages. However, partial loss-of-function situation in the worm model displayed growth retardation. In mice, although no knockout models have been established for the gene, knockout lines were generated for the *Sac3* [19] and *ArPIKfyve* [20] genes, both of which are functionally associated with the *PIKFYVE* gene. In both knockout lines, neurologic defects and juvenile or perinatal death was seen. Thus, complete loss of function of the *PIKFYVE* gene may lead to death in many organisms, possibly also in humans, which may account for the fact that all of the mutations thus far reported within the *PIKFYVE* gene, including the mutation shown in this report, are heterozygous.

No mutations have been previously reported for this disease in the Japanese population. In addition, even on a global scale, only two studies have been conducted to report nine mutations within the *PIKFYVE* gene in patients with FCD [13,15] (Table 2). This may be mostly due to the quite faint corneal phenotype with almost no disturbance in visual function in patients with this disease, even when those patients are older. We theorize that most ophthalmologists may fail to notice the subtle opacities associated with this corneal dystrophy. Moreover, there is a good chance that most patients with this disease might never visit an eye clinic complaining of symptoms associated with this disease. We imagine that the prevalence of patients with FCD might be much more common than has been recognized in previous reports, and potentially may exist in many countries.

**Table 2:** List of mutations thus far reported within the *PIKFYVE* gene in FCD patients.

Region	Nucleotide change	Amino acid change	Original description	Report
Exon 16	c.2098delA	p.Asn701ThrfsX7	2256delA	Li et al. [15]
Exon 16	c.2116_2117delCT	p.Leu706ValfsX6	2274delCT	Li et al. [15]
Intron 19	c.3619 −1G>C	p.Val1207AlafsX11	IVS19–1G→C,intron 19	Li et al. [15]
Exon 19	c.2551C>T	p.Arg851X	R851X	Li et al. [15]
Exon 19	c.2962C>T	p.Gln988X	Q988X	Li et al. [15]
Exon 19	c.3088G>T	p.Glu1030X	E1030X	Li et al. [15]
Exon 19	c.3112C>T	p.Arg1038X	R1038X	Li et al. [15]
Exon 19	c.3308A>G	p.Lys1103Arg	K1103R	Li et al. [15]
Exon 19	c.2902_2905delCCTT	p.Asp1021ThrfsX28	c.3060–3063delCCTT	Kotoulas et al. [13]
Exon 24	c.4166_4169delAAGT	p.Glu1389AspfsX16	p.Glu1389AspfsX16	This report

The notation convention of the nucleotide and amino acid changes follows the nomenclature guidelines of human genome variation society (HGVS). Note that there is a confusing situation in that the numbers of the exons and the intron of the *PIKFYVE* mutations listed in IC3D [21] appear to be incorrect, while those originally described by Li et al. [15] are correct. There is also another confusing situation in that the nucleotide number of the mutation reported by Li et al. and Kotoulas et al. [13] appears to be of mRNA, not of the coding sequence. We corrected this nucleotide number so that it is now in accordance with the nomenclature convention of the HGVS.

In summary, we show here that a novel mutation (p.Glu1389AspfsX16) causing the truncation of the *PIKFYVE* protein causes fleck corneal dystrophy in the Japanese population. We hope that this study will contribute to future investigations focusing on understanding the biochemical properties and physiologic significance of the *PIKFYVE* gene as well as elucidating the molecular pathogenesis of FCD.
